# Supplementary material for: Vitamin D Modified DSS-Induced Colitis in Mice via STING Signaling Pathway
Source: Biology (Basel). 2025 Jun 18;14(6):715. doi: 10.3390/biology14060715 (PMC12190092; doi:10.3390/biology14060715)
Supplement: Supplementary file 1 [file biology-14-00715-s001.zip › Supplementary Materials S1: 16s-report of gut microbiota/Groups/betadiv/pcoa_bray_analysis/Pcoa_bray13_boxplot_nolabel.pdf]

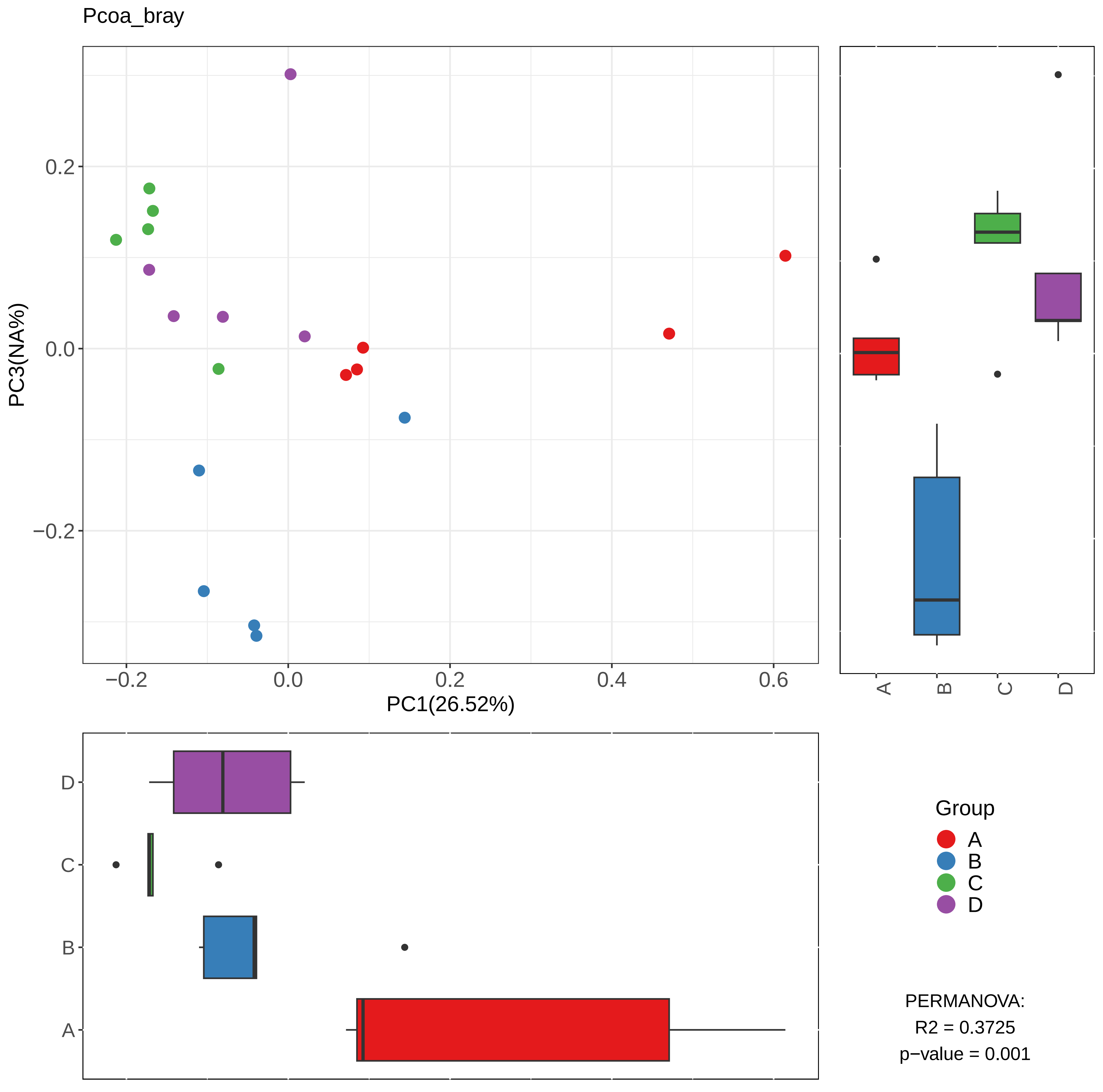
Pcoa_bray
PC3(NA%)
PC1(26.52%)
0.2
0.0
−0.2
−0.2
0.0
0.2
0.4
0.6
A
B
C
D
Group
A
B
C
D
PERMANOVA:
R2 = 0.3725
p−value = 0.001
